# CyDiv, a Conserved and Novel Filamentous Cyanobacterial Cell Division Protein Involved in Septum Localization

**DOI:** 10.3389/fmicb.2016.00094

**Published:** 2016-02-10

**Authors:** Dinka Mandakovic, Carla Trigo, Derly Andrade, Brenda Riquelme, Gabriela Gómez-Lillo, Katia Soto-Liebe, Beatriz Díez, Mónica Vásquez

**Affiliations:** ^1^Fondap Center for Genome Regulation, Universidad de ChileSantiago, Chile; ^2^Laboratorio de Ecología Microbiana y Toxicología Ambiental, Department of Molecular Genetics and Microbiology, Pontificia Universidad Católica de ChileSantiago, Chile; ^3^Laboratorio de Ecología Microbiana de Sistemas Extremos, Department of Molecular Genetics and Microbiology, Pontificia Universidad Católica de ChileSantiago, Chile

**Keywords:** filamentous cyanobacteria, cell division, septum

## Abstract

Cell division in bacteria has been studied mostly in *Escherichia coli* and *Bacillus subtilis*, model organisms for Gram-negative and Gram-positive bacteria, respectively. However, cell division in filamentous cyanobacteria is poorly understood. Here, we identified a novel protein, named CyDiv (**Cy**anobacterial **Div**ision), encoded by the *all2320* gene in *Anabaena* sp. PCC 7120. We show that CyDiv plays a key role during cell division. CyDiv has been previously described only as an exclusive and conserved hypothetical protein in filamentous cyanobacteria. Using polyclonal antibodies against CyDiv, we showed that it localizes at different positions depending on cell division timing: poles, septum, in both daughter cells, but also in only one of the daughter cells. The partial deletion of CyDiv gene generates partial defects in cell division, including severe membrane instability and anomalous septum localization during late division. The inability to complete knock out CyDiv strains suggests that it is an essential gene. *In silico* structural protein analyses and our experimental results suggest that CyDiv is an FtsB/DivIC-like protein, and could therefore, be part of an essential late divisome complex in *Anabaena* sp. PCC 7120.

## Introduction

Cyanobacteria are unique microorganisms that have been major determinants of the evolution of life on Earth as they converted our planet’s reducing atmosphere into an oxidizing one by being the initiators of oxygenic photosynthesis (Cavalier-Smith, 2010). Cyanobacteria are divided into five subsections depending on their morphologies, where I and II include unicellular coccoids and III to V involve filamentous forms. Filamentous cyanobacteria from sub-sections IV and V are considered true multicellular microorganisms because some cells in a filament can differentiate into specialized forms (Rippka et al., 1979). Heterocysts, for example, provide a suitable micro-oxic environment for nitrogen fixation, a process catalyzed by the oxygen-sensitive enzyme nitrogenase (Singh and Montgomery, 2011). Unlike unicellular forms, filamentous cyanobacterial cells remain connected even after cell division is completed and the outer membrane is continuous along the filament. Furthermore, cell communication and filament maintenance is mediated by a continuous periplasm (Flores et al., 2006). Thus, the question of how cell division occurs in filamentous cyanobacteria is intriguing.

Cell division mechanisms in bacteria have been mostly studied in model unicellular bacteria such as *Escherichia coli* (Gram-negative) and *Bacillus subtilis* (Gram-positive). The first stage is the formation of a Z-ring at the division site, strictly at mid-cell, composed of the polymerized tubulin-like protein FtsZ. The division process depends on both, location and time of assembly of the Z-ring. These processes are controlled by regulatory mechanisms that include the nucleoid occlusion and Min systems (Egan and Vollmer, 2013). Even though cyanobacteria are considered to be Gram-negative by cellular morphology (Flores et al., 2006), they have a close phylogenetic relationship with Gram-positive bacteria (Battistuzzi and Hedges, 2009). Regarding cellular division, genes from both Gram-negative and Gram-positive bacteria have homologs in cyanobacterial genomes (*ftsZ, sulA, ftsI, ftsQ, ftsW, sepF, ami*, DIPM/NlpD, *envC*, and *minCDE*) (Miyagishima et al., 2005, 2014) or have not been detected in cyanobacteria (*ftsA, zipA, zapA, ftsK, ftsB, ftsL, ftsN*) (Lutkenhaus, 2007; Marbouty et al., 2009a; Miyagishima et al., 2014), while other cell division genes seem to be exclusive of cyanobacteria (*ftn2, ftn6, alr2338* also named *sepJ*) (Koksharova and Wolk, 2002a,b; Mazouni et al., 2004). Therefore, cell division in cyanobacteria is probably different from that of the most studied Gram-positive and Gram-negative model microorganisms.

**FIGURE 1:**
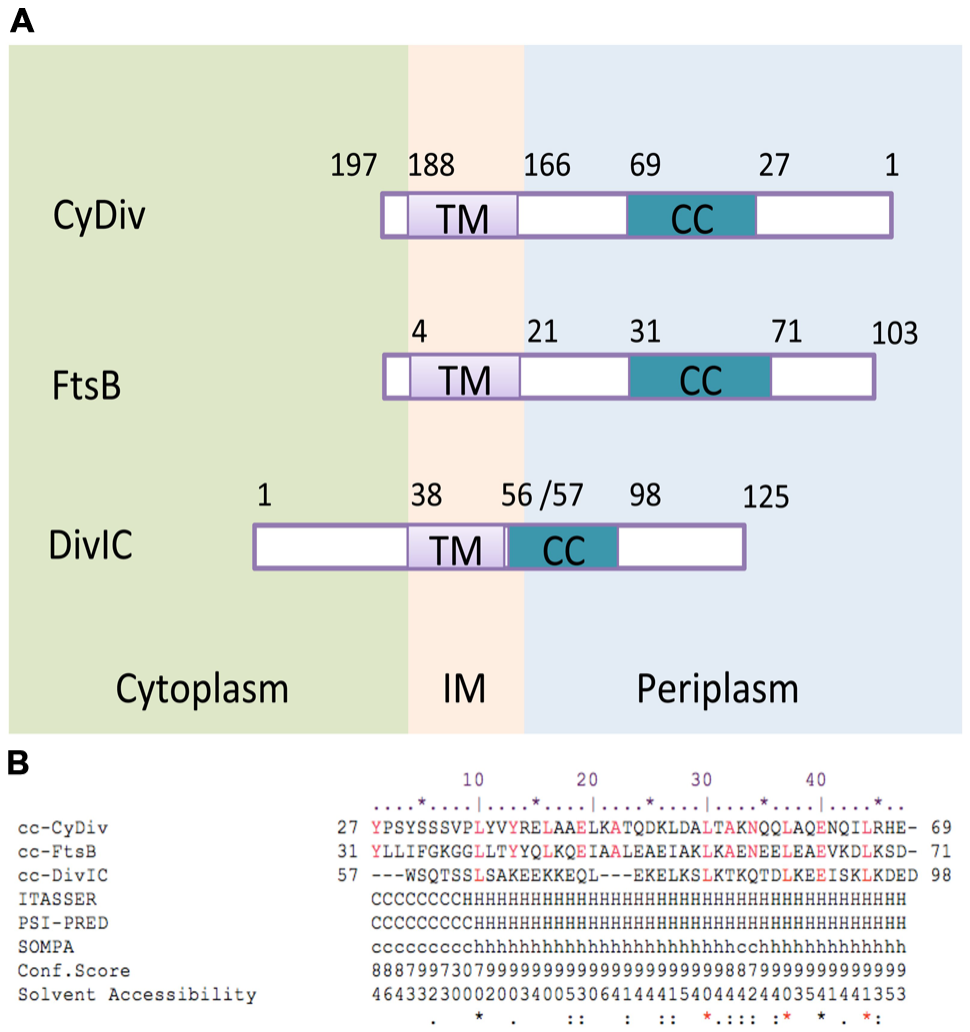
***In silico* analysis of functional and structural segments of the CyDiv (**Cy**anobacterial **Div**ision) protein. (A)** Topology comparison of “transmembrane” (TM) and “coiled-coil” (CC) domains of the CyDiv protein, and the homolog proteins FtsB of *Escherichia coli*, and DivIC of *Bacillus subtilis*. IM: Inner membrane **(B)**. Sequence comparison of the “CC” domain of CyDiv with the homologous “CC” domains of FtsB and DivIC. The secondary structure prediction analysis was performed in ITASSER, PSI-PRED, and SOMPA servers. H: Helix, C: Coil. Predicted Solvent Accessibility: Values range from 0 (buried residue) to 9 (highly exposed residue). Conserved amino acids are indicated in red.

Little information is available for specific proteins or mechanisms involved in cellular division of filamentous cyanobacteria. *Anabaena* sp. PCC 7120 (hereafter *Anabaena* PCC7120) division genes include *ftsZ, zipN* (*ftn2*), which would encode an anchor protein of FtsZ to the cytoplasmic membrane, *ftn6*, with potential function in filamentous cyanobacteria cellular differentiation and division, and the membrane associated divisome components encoded by *ftsQ, ftsW* and *sepJ* (Koksharova and Wolk, 2002a; Errington et al., 2003; Goehring and Beckwith, 2005; Harry et al., 2006; Marbouty et al., 2009b; Ramos-Leon et al., 2015). In order to unveil novel proteins involved in cellular division of filamentous cyanobacteria, we first identified genes found exclusively in these organisms (Stucken et al., 2010). One of these exclusive genes is *all2320* from *Anabaena* PCC7120, which codes for a conserved hypothetical protein. This protein bears topological similarities to DivIC, one of the proteins that localizes at the division site during cell division in *B. subtilis;* and also to its homolog in *E. coli*, FtsB (Levin and Losick, 1994; Katis et al., 1997; Errington et al., 2003; Goehring and Beckwith, 2005; Harry et al., 2006; **Figure 1**). Here, we describe the role of All2320 (hereafter named CyDiv, **Cy**anobacterial **Div**ision) in *Anabaena* PCC7120. We developed anti-CyDiv polyclonal antibodies to investigate cell localization of CyDiv and, in order to establish the potential function of this protein, we generated an *all2320* mutant strain through site-directed deletion. Our analyses of CyDiv localization and function suggest its prospective involvement in filamentous cyanobacterial cell division.

## Results

### CyDiv is Associated to the Assembly of the Divisome

Our *in silico* analyses of CyDiv (see Materials and Methods) show a predicted protein of 197 amino-acids in *Anabaena* PCC7120. This protein comprises a 42-residue “coiled-coil” (CC) region near the N-terminus (residues 27 to 69), which may allow interaction with other proteins; and a predicted “transmembrane” domain of 22 residues near the C-terminus (residues 166 to 188), that includes a leucine zipper motif (L-7L-7L) (**Figure 1**). The topology prediction indicates that the N-terminus is periplasmic, while the short C-terminal tail is cytoplasmic (**Figure 1**). CyDiv shows its highest similarity to the gamma Proteobacteria FtsB protein (16%) and to the previously mentioned conserved domains. Albeit having its “CC” domain in the opposite end, CyDiv shows its highest secondary structure similarity to *E. coli* FtsB (52%) and to DivIC (63%), its homolog in *B. subtilis*.

### CyDiv Localizes at Different Positions During Cell Division

To localize the protein in the cell, CyDiv was expressed in *E. coli* and purified, and anti-CyDiv polyclonal antibodies were generated as described in Section “Materials and Methods”. The antibodies specificity was tested by western blot analysis of *Anabaena* PCC7120 extracted proteins from the membrane fraction (Supplementary Figure S1), since the protein was not detected in the soluble fraction (data not shown). A signal corresponding to a protein slightly higher than 23 kDa was detected for *Anabaena* PCC7120, probably owed by post translational modifications produced in the protein. Less intensified and unspecific bands were also detected in the western blot, possibly due to a common post-purification degradation process or tight interaction between CyDiv–CyDiv and other proteins, which have not been yet identified but are being analyzed by our group. Also, the antibodies were evaluated against a C-truncated CyDiv protein (residues 1–180) of approximately 19 kDa expressed heterologously in *E. coli*, where a predicted sized intensified band was observed (Supplementary Figure S1). An anti-HisTag antibody was also analyzed, were we could observe the expected sized band in the truncated protein (Supplementary Figure S1).

**FIGURE 2:**
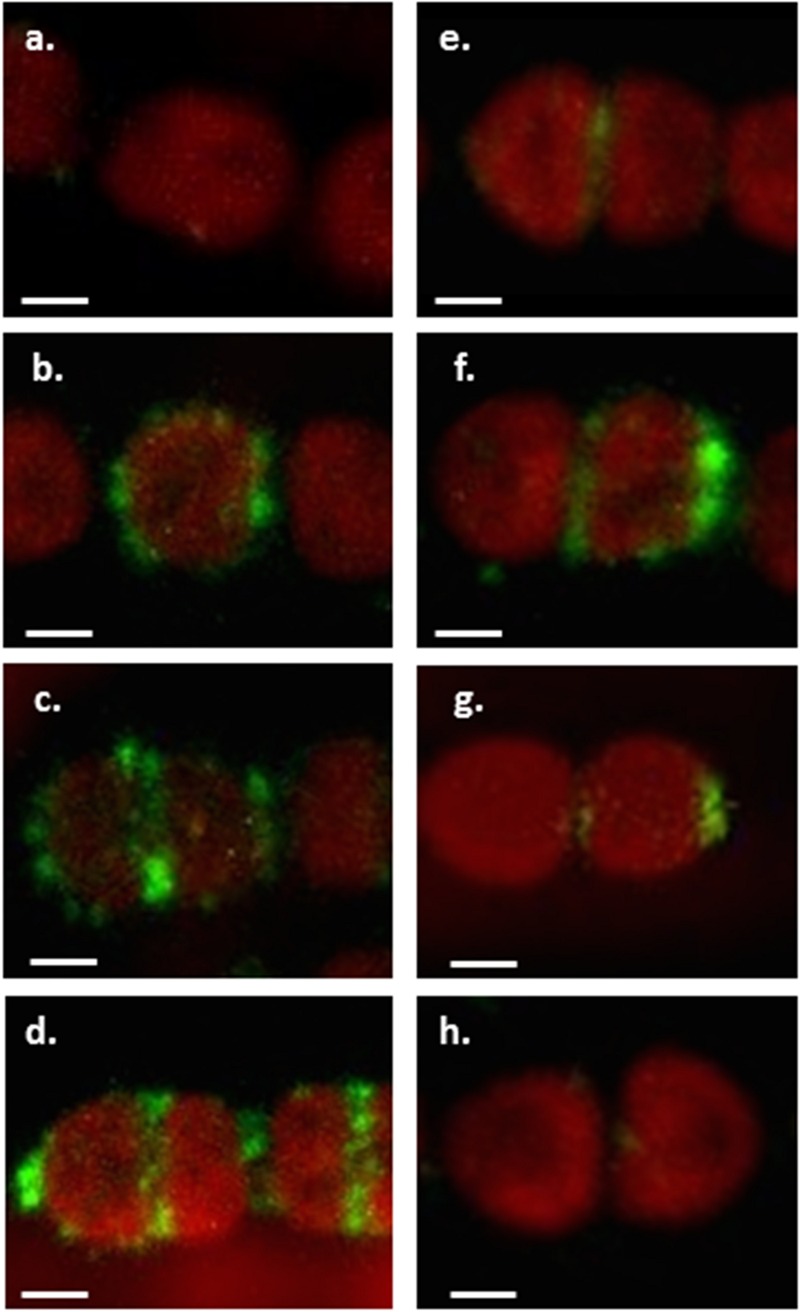
**Immunolocalization of CyDiv in *Anabaena* PCC7120.** Immunolocalization of CyDiv by confocal fluorescent microscopy. Confocal images show different immunolocalizations of the protein depending on the division state of each cell using a polyclonal anti-CyDiv antibody and as a secondary antibody, Alexa Fluor 488 goat anti-rabbit IgG (Invitrogen). The red autofluorescence observed in the cells is due to cyanobacterial chlorophyll. **(a)** Cyanobacterial chlorophyll fluorescence in average size vegetative cells not undergoing division. **(b)** CyDiv spread slightly across the cell membrane. **(c)** CyDiv localization in the membrane of elongated vegetative cells and as a very thin line at mid-cell. **(d)** CyDiv localization at the poles and in the mid-cell area of some cells of the filament. **(e)** CyDiv localization as a sharp signal at mid-cell, coinciding with the space between cells visibly constricting. **(f)** CyDiv localization as a sharp signal at mid-cell and in only one cell pole. **(g)** CyDiv localization primarily in one cell pole of small cells. **(h)** No CyDiv fluorescence observed in average size vegetative cells. Bar: 1 μm.

We labeled *Anabaena* PCC7120 filaments grown under combined nitrogen with these antibodies and analyzed around 100 filaments by confocal microscopy (**Figure 2**). We found that the pattern of CyDiv localization correlates with cell division state. CyDiv is not present in most average size vegetative cells not undergoing division (**Figure 2a**). However, in some cases, it was possible to observe that CyDiv spread slightly across the cell membrane (**Figure 2b**). In longer vegetative cells (elongated cells) CyDiv localizes in the cell membrane and as a very thin line at mid-cell (**Figure 2c**). Eventually, the protein localizes at the poles and in the mid-cell area (**Figure 2d**). Some cells that were visibly constricting show (i) a sharp signal of CyDiv at mid-cell, coinciding with the space between cells (**Figure 2e**), or (ii), a sharp signal of CyDiv at mid-cell and in only one pole of the cell (**Figure 2f**). Finally, in small cells, CyDiv is primarily located in one cell pole (**Figure 2g**). After division is completed, the protein is no longer observed (**Figure 2h**). We used the pre-immune serum to label the filaments as a control of cellular localization, which resulted negative, as expected (data not shown).

### Partially Deleted CyDiv Generates Defects in Cell Division

To evaluate the potential role of CyDiv in filamentous cyanobacteria cell division, we generated a partially segregated *all2320* mutant strain by interrupting the gene with the Streptomycin and Spectinomycin resistance cassette C.S3 (for details, see Materials and Methods). To corroborate the interruption of *all2320* with C.S3 in the mutant strain, we used all2320-1 and all2320-2 primers to amplify the region of the insertion, and all2320extCS3F and CS3rev primers set to amplify an internal 535 bp C.S3 region (Supplementary Table S1; Supplementary Figure S2). We observed the presence of two stronger bands in *all2320*::C.S3 strain using the first primer set: a 1,039 bp that indicated the absence of the cassette insertion, and also a 3,101 bp that indicated that the strain was not fully segregated. The amplification of the second primer set showed the expected 535 bp CS.3 cassette amplification, confirming that not all the copies of the cyanobacterial chromosome had *all2320* disrupted.

**FIGURE 3:**
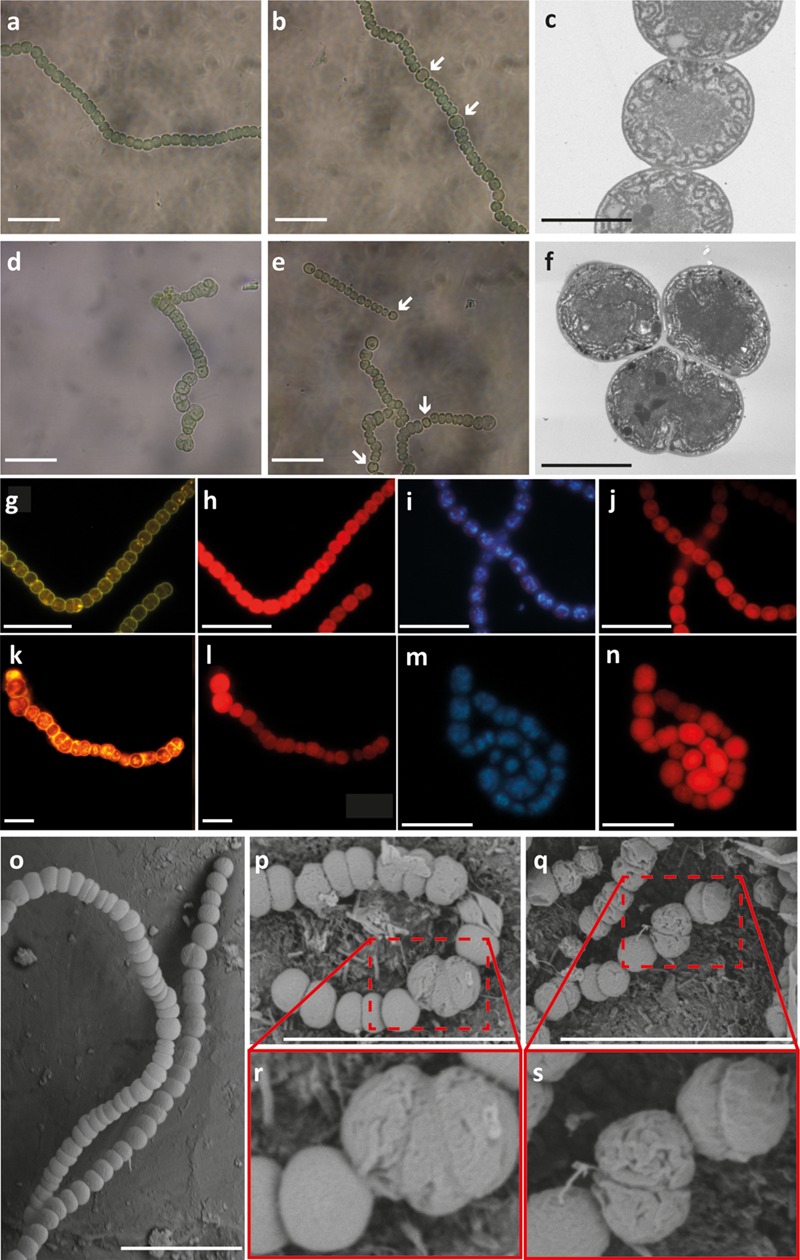
**Phenotype of *Anabaena* PCC7120 and *all2320*::C.S3 strains.** Light microscopy of *Anabaena* PCC 7120 (wild type, wt) and *all2320*::C.S3 cultures grown with combined nitrogen (**a,d**, respectively) and in the absence of combined nitrogen (**b,e**, respectively). White arrows indicate heterocysts. Transmission electron microscopy of wt and *all2320*::C.S3 strains (**c,f**, respectively). Cytoplasmic membranes of wt and *all2320*::C.S3 strains stained with FM1-43FX^®^(**g,k**, respectively). Micrographs showing the autofluorescence of the same previous samples in wt and *all2320*::C.S3 strains (**h,l**, respectively). DNA DAPI staining of wt and *all2320*::C.S3 strains (**i,m**, respectively). Micrographs showing the autofluorescence of the same previous samples in wt and *all2320*::C.S3 strains (**j,n,** respectively). Scanning electron microscopy (SEM) of filaments from wt **(o)** and *all2320*::C.S3 strains **(p**–**s)**. White bar: 10 μm, gray bar: 2 μm.

The previous result was concordant with our observation of two different types of filaments in the *all2320*::C.S3 strain grown in the presence or absence of combined nitrogen: most of the filaments (around 70%) exhibited a wild type phenotype, showing no differences in filament length or cell size compared to *Anabaena* PCC7120 (**Figures 3a,b**). However, fewer filaments revealed an altered cell division compared to the wild type strain (mutant phenotype filaments) (**Figures 3d,e**). By light microscopy, these altered filaments displayed a misplaced septum formation during cell division that dramatically affects cell morphology (**Figures 3d,e**). Nevertheless, when grown under nitrogen deprivation, both types of *all2320*::C.S3 filaments developed normal heterocyst differentiation and pattern formation when compared to the wild type strain (**Figures 3b,e**, respectively), suggesting that *all2320* is not regulated by nitrogen. To confirm this hypothesis, we performed a transcriptional analysis of *all2320* in *Anabaena* PCC7120 cultures grown in the presence and absence of combined nitrogen, using internal primers qall2320 F and qall2320 R (Supplementary Table S1), as described in Section “Materials and Methods.” As an experimental control, we analyzed the transcription of *hglD* (GenBankAAA93154.1) (primers qhglD F/qhglD R, Supplementary Table S1), under the same conditions. This gene is responsible for the glycolipid synthase protein, essential for heterocyst development (Bauer et al., 1997). As expected, *hglD* was only overexpressed in cultures grown under nitrogen deprivation for 24 and 48 h, when there were a large number of heterocysts per filament (Supplementary Figure S3). Regarding *all2320*, we did not observe transcription induction or repression of the gene under either condition (Supplementary Figure S3). These results indicate that *all2320* transcription is not regulated by nitrogen metabolism and suggest that CyDiv is necessary for a biological process that probably operates independently of the nitrogen source.

When analyzing the *all2320*::C.S3 mutant phenotype filaments by TEM, it was evident that the forming septum showed an atypical location in some of the cells (**Figures 3c,f**). Therefore, it is possible to presume an unusual positioning of the Z-ring in vegetative cells of these filaments. This phenotype suggests that early cell division proteins are not affected in the mutant phenotype filaments since the septum-like structures can be formed.

To analyze the continuity and stability of the cell inner and outer membranes in *all2320*::CS3, around 150 filaments were stained with the FM^®^1-43FX dye that binds to phospholipids bilayer membranes (Molecular Probes^TM^) (Schneider et al., 2007). We noticed a brighter labeling of the molecular dye into the *all2320::CS3* mutant cells when compared to wild type filaments (**Figures 3g,k**). We performed the membrane staining of *all2320::CS3* mutant phenotype filaments several times, always observing the same diffusion of the molecular dye into the cytoplasm. This result suggests membrane instability in these cells as (Nurnberg et al., 2014) and (Zeineddine et al., 2015) have previously reported this type of leakage in cells with membrane perturbations. Due to “minicell” formation in *all2320::CS3* mutant phenotype filaments, we evaluated the DNA distribution by DAPI staining. This staining showed a normal DNA localization (**Figures 3i,m**), thus quantitative methods are needed to identify if *all2320::CS3* mutant phenotype filaments show defects in DNA segregation. The autofluorescence due to chlorophyll was observed normal for the *all2320::CS3* mutant cells when compared to wild type filaments (**Figures 3h,j,l,n**). Based on these observations, we performed SEM to test if the cell envelope structure of *all2320::CS3* was affected. The results revealed that the cells in *all2320*::CS3 mutant phenotype filaments have delocalized division planes (**Figures 3p,r**) and show wrinkled cell surfaces when compared to the wild type strain (**Figures 3p–s,o**). Finally, we observed that after approximately 48 h of growth in the presence and absence of combined nitrogen, both in liquid and in solid medium, the mutant phenotype filaments of *all2320*::C.S3 began to disappear from the culture (Supplementary Figure S4).

## Discussion

FtsB and DivIC belong to the subcomplexes FtsQ-FtsL-FtsB and DivIB-FtsL-DivIC, which are involved in the late recruitment of proteins of the divisome in *E. coli* and *B. subtilis* respectively, including proteins required for cell wall synthesis (Katis et al., 1997; Buddelmeijer and Beckwith, 2004; Harry et al., 2006). Our *in silico* analyses showing the highest similarity of CyDiv with FtsB and DivIC are the first evidence that suggests that CyDiv may be a new component in the cyanobacterial divisome, and that it is likely to interact with other protein(s) during filamentous cyanobacteria cell division. Moreover, when analyzing the protein localization (**Figure 2**), we observed that the pattern of CyDiv localization correlated with cell division state. This is not new when observing other proteins involved in the bacterial cell division process, where the spatial localization of these proteins include (i) one or both cell poles in rod-shaped cells; (ii) mid-cell in rod-shaped and spherical cells; (iii) along the long axis of rod-shaped cells; (iv) in specific structures such as stalks or endospores, and (v) oscillatory localization (Treuner-Lange and Sogaard-Andersen, 2014). Nevertheless, the localization of CyDiv during cell division is not exactly the same to what has been described for cellular division proteins in other microorganisms. Thus, the determination of CyDiv localization dynamics, could have given additional insights in its role in multicellularity and cellular division. First attempts using GFP fusion protein were unsuccessful although fusion at both ends of the protein were constructed. It is important to mention that we did not observe any labeling when using the pre-immune serum to mark the filaments as a control of cellular localization. We could not label CyDiv mutant phenotype filaments as an antibody labeling control because these filaments lysed before we could perform this assay.

According to the observation of all the phenotypic results obtained from the mutant strain, and bearing in mind that *all2320*::C.S3 is not a fully segregated strain, we propose that probably the filaments presenting the mutant phenotype displayed a lethal phenotype caused by an apparent essentiality of the protein, while wild type phenotype filaments were probably due to a not fully segregated mutation of *all2320* or no mutation at all in the copies of their chromosomes. It is imperative to mention that although there is a need to develop a 100% knockout of the protein in order to really evaluate the essentiality of the protein, the variable polyploidy of *Anabaena* sp. PCC 7120 makes it a difficult task. In fact, we performed the CyDiv mutation process twice (biological replicates), and we could never obtain fully segregated clones. Nevertheless, our group is working in the development of an inducible CyDiv deletion strain and in the generation of strains with deletion of some motifs of the protein, in order to try to resolve this issue in the future.

In the filaments of *all2320*::C.S3 that showed a mutant phenotype, TEM analysis evidenced an atypical location of the forming septum (**Figures 3c,f**). Therefore, it is possible to presume an unusual positioning of the Z-ring in vegetative *all2320*::C.S3 mutant cells in these filaments. This phenotype suggests that early cell division proteins are not affected in the mutant strain since the septum-like structures can be formed. FM^®^1-43FX staining and SEM observations imply that CyDiv could be involved in membrane stability, suggesting that the cell wall is also affected at such level that the osmotic pressure causes cell lysis. A recent study in *E. coli* (Tsang and Bernhardt, 2014) supports a model in which the FtsQLB protein complex would function as part of a sensing mechanism that promotes the onset of the cell wall remodeling processes needed for the initiation of cell constriction after divisome assembly. The participation of FtsB in the initiation of cell constriction could explain the extreme damage observed in the cell wall of the CyDiv mutant strain. Furthermore, the mutant phenotype observed by SEM is similar to that of the triple mutant AmiABC, peptidoglycan (PG)-cleaving proteins in *E. coli* (Heidrich et al., 2001). This triple mutation causes the formation of “minicells” between long filaments and a tendency to cell wall damage in *E. coli*. Therefore, we suggest that there is a link between the cytosolic and periplasmic divisome elements involved in the positioning of the Z- ring and the synthesis of the cell wall, with consequent hydrolysis of PG in *Anabaena* PCC7120. In this way, CyDiv seems to be involved in a crucial role in stabilizing the cell wall during cell elongation, perhaps in association with other proteins that have not been identified yet.

**FIGURE 4:**
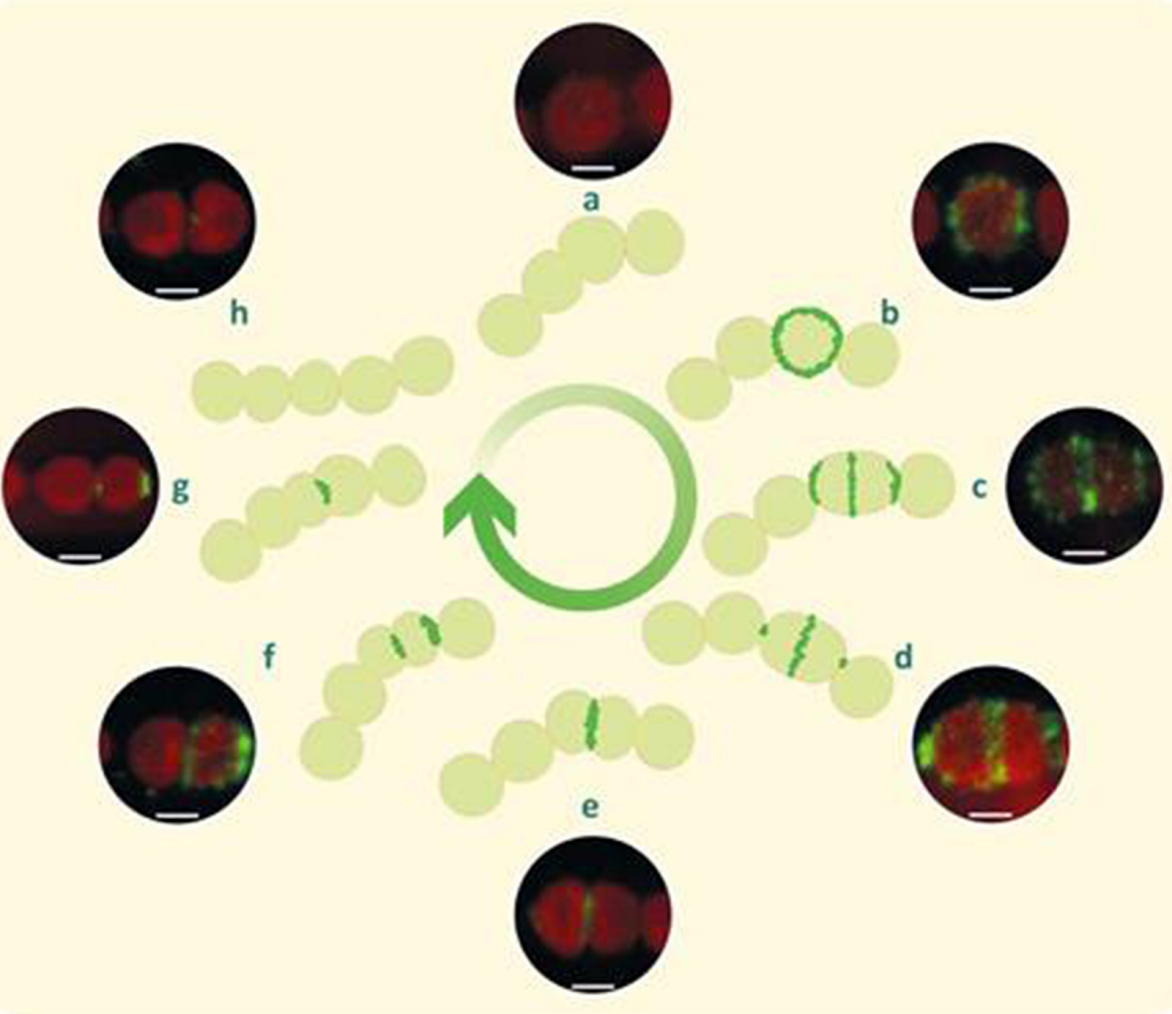
**Model of cellular localization of CyDiv in *Anabaena* PCC7120 based on immunolocalization.** Initially, CyDiv is localized in the cell membrane **(b)**, and then in the membrane and septum of growing and dividing cells **(c)**. Afterward, it localized mainly in the septum and to a lesser extent at the poles **(d)**. Later, CyDiv localizes only in the septum **(e)**. Subsequently, CyDiv remains in the septum but it also localizes in the pole of one of the daughter cells **(f)**. Finally, the protein is located only at the pole of one of the daughter cells **(g)**. The initial and final steps are represented in **(a,h)**, respectively. Bar: 1 μm.

Consequently and mainly based on the immunofluorescence observations and the *all2320*::CS3 mutant phenotype filaments, we propose a model for CyDiv dynamic and distinctive localization during cellular division (**Figure 4**). Initially, CyDiv is expressed and then anchored to the cell membrane in some of the cells that present average size (**Figure 4b**). Subsequently, in the elongated cells, CyDiv remains localized in the cell membrane and also in mid-cell, possibly interacting with the Z-ring (Sakr et al., 2006; **Figure 4c**); sometimes, there is also strong labeling in the cell poles (**Figure 4d**). Next, CyDiv is located only in the mid-cell constriction sites (**Figure 4e**), suggesting that CyDiv interacts with divisome elements, but its particular function is not associated to Z-ring assembly at early stages of cell division. TEM to the *all2320*::CS3 mutant phenotype filaments indicate that CyDiv affects the constriction ring position (**Figure 3f**). Therefore, we propose that CyDiv migrates to the pole in one of the two daughter cells, as has been described for cell division proteins PopZ and TipN of *Caulobacter crescentus*, which show a unipolar to bipolar transition during the cell division (Huitema et al., 2006; Vecchiarelli et al., 2012). Thus, the final localization of CyDiv would be only in one cell pole of one of the daughter cells (**Figure 4g**). In the case of CyDiv, it is risky to define whether this protein is involved in the recognition of one specific pole, either new or old, as is the case of TipN (in *C. crescentus*) or ActA (in *Listeria monocytogenes*). Therefore, experiments of co-localization of CyDiv with other cell division proteins are being developed by our group in order to better understand the “mother” cell issue in filamentous cyanobacteria.

Bacterial cell polarity can be explained, according to the literature, by three hypotheses: “Diffusion and capture” (Rudner and Losick, 2010), “Protein localization by matrix-dependent, self-organizing ParA/MinD ATPases” (Thanbichler and Shapiro, 2006) and “Protein localization by small GTPases” (Treuner-Lange and Sogaard-Andersen, 2014). The closest hypothesis fitting the unusual localization pattern of CyDiv is “Diffusion and capture”. This hypothesis includes three aspects: protein-protein interaction, where one protein diffuses through the cytoplasm until it recognizes another polar protein, such as the MinC-D and MinE interaction (Bramkamp et al., 2008); protein affinity to the negative curvature of cell poles such as DivIVA, which preferentially assemble to membranes with negative curvature (Lenarcic et al., 2009); and affinity for polar features of the cell envelope, for example ProP of *E. coli*, which localizes in the cell pole in a cardiolipin-dependent manner (specific phospholipids) (Romantsov et al., 2007). The protein-protein interaction hypothesis fits with our proposed model described in **Figure 4**, due to the changing localization of CyDiv through cell division stages in *Anabaena* PCC7120, suggesting a coordinated movement until an asymmetric polar position is achieved at the end of cell division, possibly by recognition of other polar proteins involved in this process. On the other hand, multimerization of a protein controlled by cell cycle events that result in a unipolar to bipolar transition localization also resembles the proposed model of CyDiv dynamics. This is the case of PopZ protein of *C. cressentus.* The multimerization process of PopZ is coupled to the asymmetric distribution of other proteins, including ParA, ParB (proteins involved in plasmid and chromosome segregation, positioning of cell division site and cytoplasmic and polar protein complex) and cell cycle regulators of the G1-to S transition (Vecchiarelli et al., 2012). Moreover, PopZ multimerization includes temporal and spatial localization events during the assembly in regions of low DNA content. This location is confined to one pole of the mother cell that will be transmitted to the old pole of the daughter cells. These events are related to the cell division stage and to the “stochastic multimerization” that leads to the migration of these proteins to one cell pole (Hakkinen et al., 2013; Laloux and Jacobs-Wagner, 2013). Hence, taking together all these information, the localization pattern of CyDiv implies its participation in the cell division process of *Anabaena* PCC7120.

## Conclusion

CyDiv localization, partial mutant phenotype and its topological characteristics strongly suggest that this protein is a filamentous cyanobacteria cell division protein. The suggestion that its mutation is lethal implies that it is essential for this process, where it is involved in Z-ring positioning at mid-cell, as well as in negative FtsZ polymerization (like the Min system and nucleoid occlusion). Intriguingly, the unusual localization of CyDiv in one cell pole does not resemble the localization of these other proteins. Nevertheless, due to topological similarities to the essential proteins DivIC and FtsB, which are part of the sensing mechanism that promotes cell wall synthesis (Tsang and Bernhardt, 2014), we propose that CyDiv is part of an essential late divisome complex in *Anabaena* PCC7120. Additionally, scarce information is available for specific proteins or mechanisms involved in cellular division of filamentous cyanobacteria. The best studied genes in *Anabaena* sp. PCC 7120 are: *ftsZ, zipN* (*ftn2*), *ftn6, ftsQ, ftsW*, and *sepJ* (Koksharova and Wolk, 2002a; Errington et al., 2003; Goehring and Beckwith, 2005; Harry et al., 2006; Marbouty et al., 2009b; Ramos-Leon et al., 2015). It is important to notice that some of these genes seem to be exclusive of cyanobacteria: *ftn2, ftn6*, and *sepJ*. Those exclusive genes do not have homologies in Gram-negative or Gram-positive bacteria, thus they seem relevant for the cellular division process in cyanobacteria. Hence, if we know that the role of most cellular division proteins in these microorganisms is preliminary; the division in filamentous cyanobacteria is quite different to what has been usually observed in model microorganisms; and that CyDiv is an exclusive filamentous cyanobacteria protein, is not rare to discover that CyDiv has a novel cellular division location/function in these distinctive multicellular microorganisms. Therefore, it is crucial to identify the proteins that could be interacting with CyDiv (given its “CC” domain) to better understand its precise function in the division process of filamentous cyanobacteria. Our results imply that cell division in *Anabaena* PCC7120 is likely a combination of features that have been described in Gram-positive and Gram-negative bacteria, but that also has novel components like CyDiv that help to elucidate the intriguing mechanisms behind the distinctive division process observed in these multicellular microorganisms.

## Materials and Methods

### Growth Conditions

*Anabaena* PCC 7120 and mutant strains were grown in BG11 medium (supplemented with NaNO_3_) or BG110 medium (N-free medium) at 30°C in the light (25 to 75 μE m^-2^ s^-1^), in shaken (90 to 100 rpm) liquid cultures or in medium solidified with 1% Difco agar, except for samples taken from cultures used for rt-PCR. These samples were grown in MLA medium according to (Castro et al., 2004), with the addition of 2 mM NH_4_Cl as the nitrogen source instead of NaNO_3_ (MLAn), or free of combined nitrogen (MLA0) in liquid cultures at 25°C in the light (25 to 75 μE m^-2^ s^-1^).

### Alignments

CyDiv analysis from local alignment was performed by “domain enhanced lookup time accelerated BLAST” (Delta-blast) for long sequences using a subset of NCBI’s Conserved Domain Database (CDD).

### Construction of Mutants

DNA was isolated from *Anabaena* PCC7120 by the CTAB method described by (Wilson, 2001). DH5α and HB101 *E. coli* strains were used for plasmid constructions and for conjugations with *Anabaena* PCC7120, respectively, and were grown in LB medium and supplemented, when it was appropriate, with antibiotics at standard concentrations. The *all2320* gene was interrupted in a central region introducing a C.S3 cassette (Sm^R^/Sp^R^) (Elhai and Wolk, 1988b) using all2320-1/all2320-3 and all2320-4/all2320-2 primers (Supplementary Table S1). The mutation was introduced by interrupting the gene with C.S3 because by genetic context analysis, *all2320* is monocistronic (*all2320* transcription is in the same direction as *gln*B, which was demonstrated to be monocistronic; Paz-Yepes et al., 2009), thus this mutation strategy should not generate a polar transcriptional effect. The construction was assembled in the pMBL-T vector (Dominion MBL, Spain), sequenced and inserted into Cargo plasmid pRL278 (Km^R^/Neo^R^) generating the pCSD10 vector. Conjugation of *Anabaena* PCC7120 with *E. coli* HB101 carrying the plasmid pCSD10 with methylation plasmid pRL623 (Cm^R^) and *E. coli* HB101 with conjugal plasmid pRL443 was achieved as described (Elhai and Wolk, 1988a), with selection for resistance to Sm/Sp. After conjugation, Sm^R^/Sp^R^ clones were spread on BG11 medium supplemented with 5% sucrose (Cai and Wolk, 1990), and individual Suc^R^ colonies were checked by PCR looking for clones that had *all2320* interrupted by C.S3. Selected clones were studied by PCR using primers all2320-1/all2320-2 and primers *all2320* extCS3F/CS3rev to analyze the presence of C.S3 in *all2320*. The mutant strain was named *all2320*::C.S3.

### Real Time RT-PCR

RNA was isolated from *Anabaena* PCC7120 using RNeasy Plant Mini Kit (Qiagen Sciences) according to the manufacturer’s instructions. 15 ml samples for RNA extraction were taken from cultures grown during 0, 3, 6, 12, 24, and 48 h in MLAn or MLA0. Integrity of RNA was determined in 1% agarose, 86% DEPC, MOPS 1X, and formaldehyde 450 mM gels run at 7 V/cm during 3 h and stained with ethidium bromide. Ten microgram of RNA from each sample were treated with DNAse using the TURBO DNA-free Kit (Ambion) according to the manufacturer’s instructions. One microgram of the treated RNA was retrotranscribed using the ImProm-II Reverse Transcription System (Promega) with random primers and according to the specifications of the manufacturer. For real time RT-PCR amplification and quantification (Light Cycler 480, Roche), the LightCycler 480 SYBR Green I Master (Roche) kit was used following the instructions from the manufacturer. To normalize the transcripts abundance, *rnpB* (RNA portion of ribonuclease P) amplification was used as a reference gene (Vioque, 1997). To calculate gene expression fold induction (N_2_/NH_4_^+^), we used the mathematical model for relative quantification in real time rt-PCR described by (Pfaﬄ, 2001). As experimental controls for gene transcription we used *hglD*. Also, vegetative cells and heterocysts of 100 filaments of each biological replicate were counted in each condition. The average number of heterocyst over the total number of cells of the three biological replicates at all time points were averaged and statistically compared by ANOVA test.

### Antibody Generation

Polyclonal antibody generation against CyDiv was carried out by rabbit immunization with synthetic peptides. Selection of antigenic peptides was based on the hydrophobicity analysis method (Hopp and Woods, 1981). The peptide 112-cPVVPEAPSSKNRRT-125 was synthesized and inoculated into two New Zealand rabbits with boosts at 14, 35, and 56 dpi Polyclonal anti CyDiv antibodies were affinity purified and lyophilized by GenScript^®^. The antibody specificity was tested by Western Blot analysis of *Anabaena* PCC7120 extracted proteins in the presence of combined nitrogen Also, the antibodies were evaluated against a truncated CyDiv protein of approximately 19 kDa expressed heterologously in *E. coli.*

### SDS/PAGE and Immunoblotting

Protein extracts and purified C-truncated All2320 were separated with SDS/PAGE in a 15% polyacrylamyde gel and stained with colloidal coomassie blue G250. Following, proteins were transferred to PVDF membranes, blocked with 5% BSA in 1X TS buffer (blocking buffer) for 1 h at room temperature, then incubated with primary antibodies diluted 1:1000 in blocking buffer and washed three times with TS-T (0.005% Tween 20) buffer. Bound antibodies were detected using Alkaline Phosphatase goat anti-rabbit IgG (Invitrogen) (1:5,000 diluted in blocking buffer). Control experiments were performed with pre-immune serum. Proteins were detected using the Alkaline Phosphatase Conjugate Substrate Kit (BioRad).

### Immunofluorescence

A 50 μL cyanobacterial culture (OD750 nm 0.3) was spread on a poly-lysine microscope slide (Sigma–Aldrich, USA) and dried for 20 min at 50°C. The sample spots were fixed in 70% ethanol (Merck) for 30 min at –20°C. The slides were air-dried and subsequently washed three times with PBS 0.1% tween 20 for 2 min. Then, the spots were blocked with PBS 3% BSA (bovine serum albumin) 0.1% tween 20 for 15 min at room temperature. The slides were incubated with the anti All2320 primary antibody (1:1,000 dilution in PBS 3% BSA 0.1% Tween 20) for 1.5 h at 4°C, in a moisture chamber. After washing the excess serum with PBS 0.1% tween 20 three times for 2 min, the spots were incubated with the secondary antibody (Alexa Fluor488 goat anti rabbit IgG) in PBS, final concentration 10 μg/ml (Invitrogen) for 45 min at 4°C, in a moisture chamber. Afterward, spots were washed again, 20 μl of Prolong antifade reagent (Invitrogen) were added, a cover slip was placed over the spot and a sealed. The slides were visualized with a Fluoview FV1000 Confocal Microscope. Alexa Fluor^®^ 488 was excited at a wavelength of 495 nm and emitted the fluorescence at 509 nm. To visualize autofluorescence due to chlorophyll, samples were excited using 510 nm irradiation and fluorescent emission was monitored at 590 nm.

### Microscopy

For standard light microscopy (Nikon Eclipse TS100), culture samples were harvested after 48 h of growth at 30°C in the light. For TEM (Philips Tecnai 12 at 80 kV accelerating voltage), samples were concentrated by centrifugation and the pellets were fixed 4 h in 3% glutaraldehyde in a 0.134 M sodium cacodylate buffer (pH 7.2) at room temperature. Cells were washed overnight with the sodium cacodylate buffer at 4°C, and post-stained 1 h with osmium tetroxide 1% (w/v). Samples were washed three times for 10 min with distilled water, stained 1 h with uranyl acetate 1% (w/v) and washed again. Samples were dehydrated in an acetone series [30–100% (v/v), pre-embedded overnight in an EPON^TM^ resin–acetone 1:1 solution, embedded 6 h in pure EPON^TM^ resin (London Resin, Reading, UK], and finally polymerized for 24 h at 60°C. Samples were sectioned with a ultramicrotome (Sorvall MT5000, Norwalk, CT, USA), and mounted on copper grids for examination and photographed with camera CCD Megaview G2, Olympus Soft Imaging Solutions (Johan-Krane-Weg 39, D-48149 Münster). For SEM (HITACHI TM3000 at 15 kV accelerating voltage), culture samples were harvested by centrifugation and fixed in 3% glutaraldehyde in a 0.134 M sodium cacodylate buffer (pH 7.2) at room temperature. Cells were washed overnight with the sodium cacodylate buffer at 4°C, and post-stained 1 h with osmium tetroxide 1% (w/v). Fixed samples were filtered on Millipore GS filters and dried at critical point in liquid CO_2_. Finally, the samples were coated with gold. For epifluorescence microscopy (Nikon Labophot-2), 20 μL of culture samples were used for both, cell membrane staining with FM^®^1-43FX (Molecular Probes^TM^) using 5 μL of a 5 μg/ml dye working solution according to the manufacturer’s protocol, and for nucleic acid staining with DAPI (Life Technologies) as the mounting solution.

## Author Contributions

DM, experimental design, performed experiments, discussion, writing. CT, performed experiments, writing, discussion. DA, performed experiments, writing, discussion. BR, performed experiments, discussion, writing. GL, performed experiments

## Conflict of Interest Statement

The authors declare that the research was conducted in the absence of any commercial or financial relationships that could be construed as a potential conflict of interest.
