# Supplementary material for: CyDiv, a Conserved and Novel Filamentous Cyanobacterial Cell Division Protein Involved in Septum Localization
Source: Front Microbiol. 2016 Feb 10;7:94. doi: 10.3389/fmicb.2016.00094 (PMC4748335; doi:10.3389/fmicb.2016.00094)
Supplement: Supplementary file 1 [file Data_Sheet_1.DOCX]

Supplementary Material

**CyDiv, a conserved and novel filamentous Cyanobacteria cell division protein involved in septum localization.**

**Dinka Mandakovic^1,3^, Carla Trigo^1^, Derly Andrade^1^, Brenda Riquelme^1,4^, Gabriela Gómez-Lillo^1^, Katia Soto-Liebe^1,4^, Beatriz Díez^2^, Mónica Vásquez^1,*^**

^1^Laboratorio de Ecología Microbiana y Toxicología Ambiental, Department of Molecular Genetics and Microbiology, Pontificia Universidad Católica de Chile, Santiago, Chile.

^2^Laboratorio de Ecología Microbiana de Sistemas Extremos, Department of Molecular Genetics and Microbiology, Pontificia Universidad Católica de Chile, Santiago, Chile.

^3^Fondap Center for Genome Regulation (CGR), Avenida Blanco Encalada 2085, Santiago, Chile.

^4^Laboratorio de Biología Molecular y Biotecnología Vegetal, Department of Molecular Genetics and Microbiology, Pontificia Universidad Católica de Chile, Santiago, Chile.

* **Correspondence:** Dr. Mónica Vásquez, Laboratorio de Ecología Microbiana y Toxicología Ambiental, Department of Molecular Genetics and Microbiology, Pontificia Universidad Católica de Chile, Av. Libertador Bernardo O’Higgins 340, Santiago, Chile.

[mvasquez@bio.puc.cl](mailto:mvasquez@bio.puc.cl)

## Supplementary Figures

**
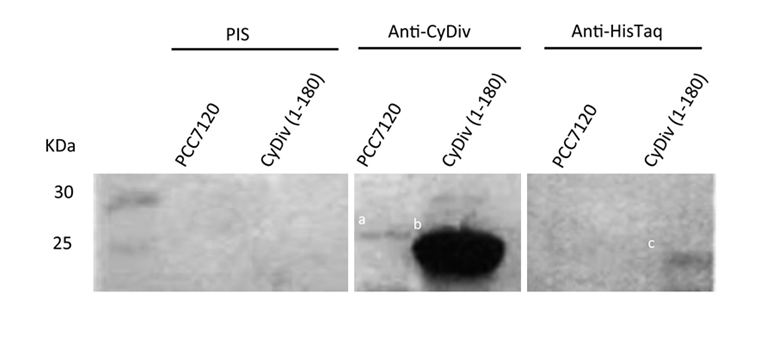
**

**Supplementary Figure 1. Western Blot analysis.** Western Blot analysis using an anti-CyDiv antibody, pre-immune serum (PIS) and anti HisTag antibody in total protein extracts of *Anabaena* PCC7120 and purified C-truncated CyDiv (residues 1-180). (a) Band corresponds to the expected size of CyDiv in *Anabaena* PCC7120 (23 kDa). (b and c) Bands correspond to the size of C-truncated CyDiv (residues 1-180) (19 kDa).


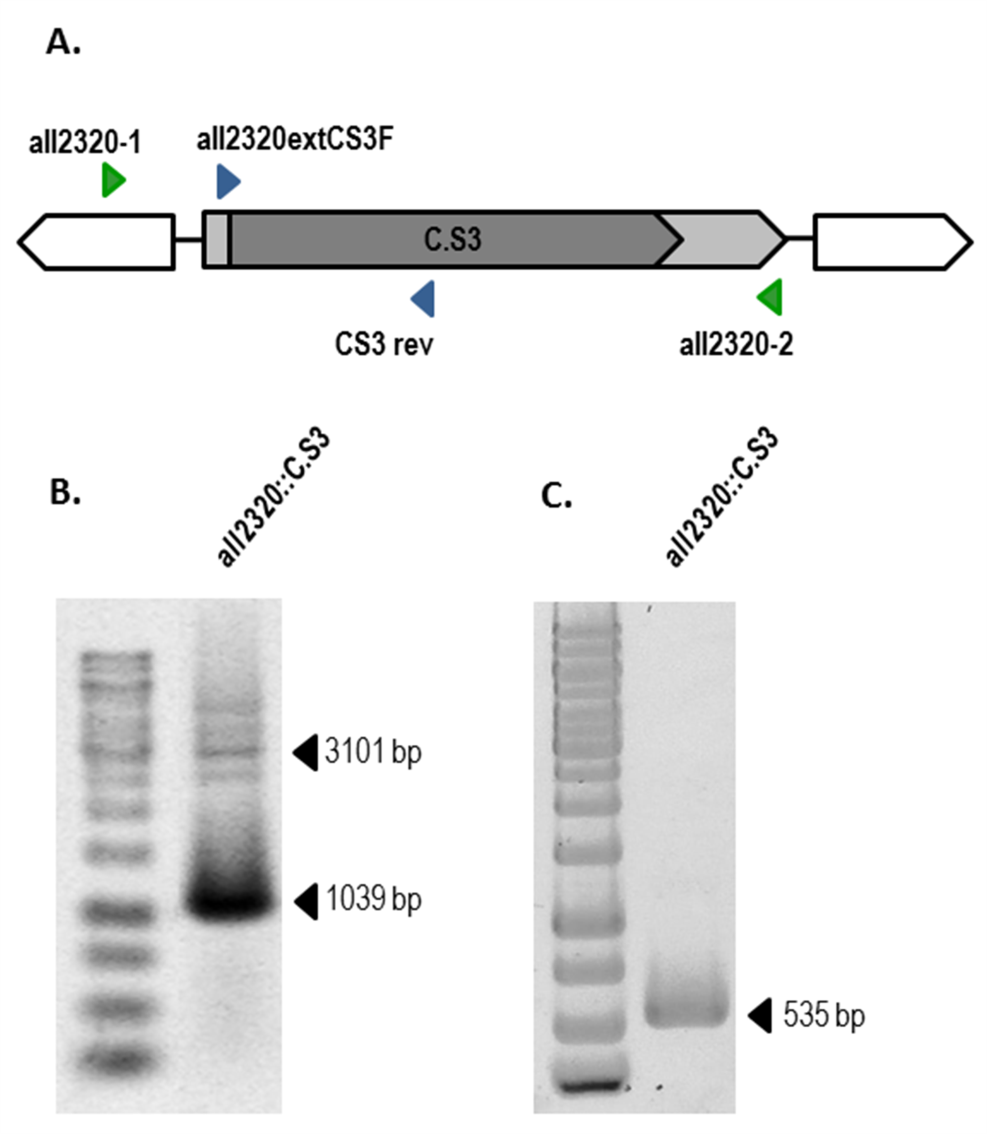


**Supplementary Figure 2. Molecular analysis of mutant segregation of *all2320::C.S3.* A**. Genetic context of *all2320* in *Anabaena* PCC7120. Arrows represent the zones where primers were designed to generate *all2320::C.S3.* **B.** Electrophoresis gel of PCR products using primers all2320-1 and all2320-2, and using DNA from *all2320::C.S3* as template*.* Arrows point to 1,039 pb and 3,101 pb bands, indicating no full segregation of the mutant strain. **C.** Electrophoresis of PCR products using primers all2320extCS3F and CS3rev, and using DNA from *all2320::C.S3* as template*.* Arrow points to a 535 pb band, indicating that some chromosomes of the cyanobacterium were mutated in *all2320*::C.S3.


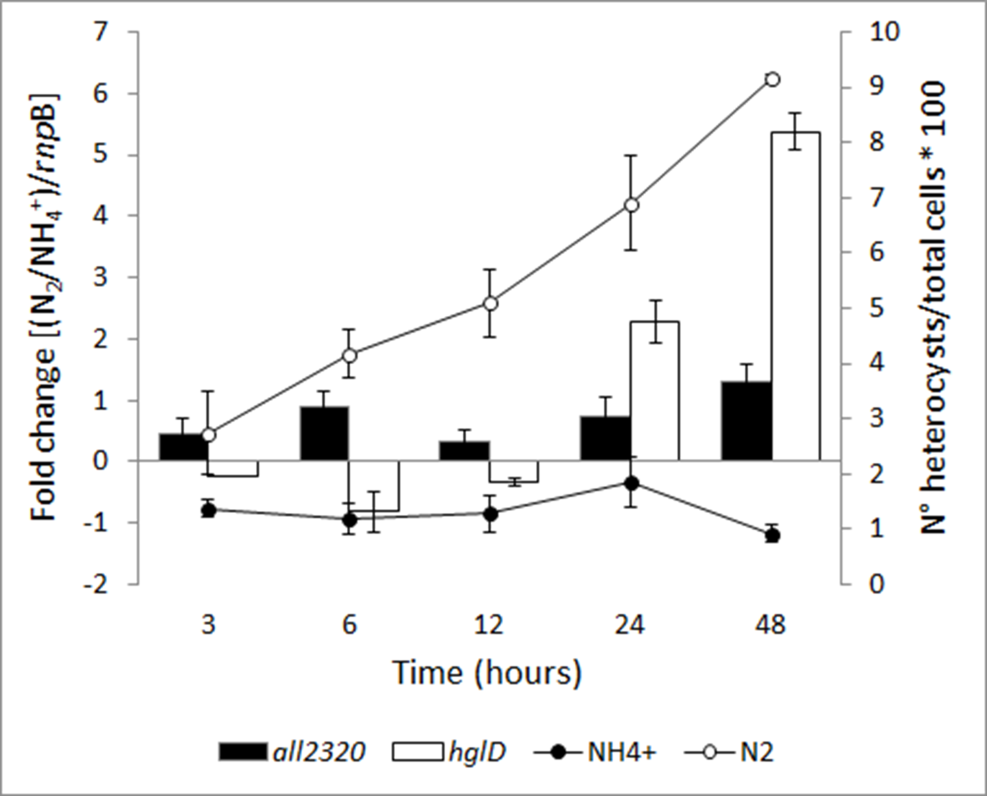


**Supplementary Figure 3. Real time rt-PCR** **analyses.** Relative transcript abundance analysis of *all2320* and control gene *hglD* and number of heterocysts per total cells. Filament samples and RNA were isolated from cultures of *Anabaena* PCC7120 grown in the presence or absence of combined nitrogen for the times indicated (in hours).

**
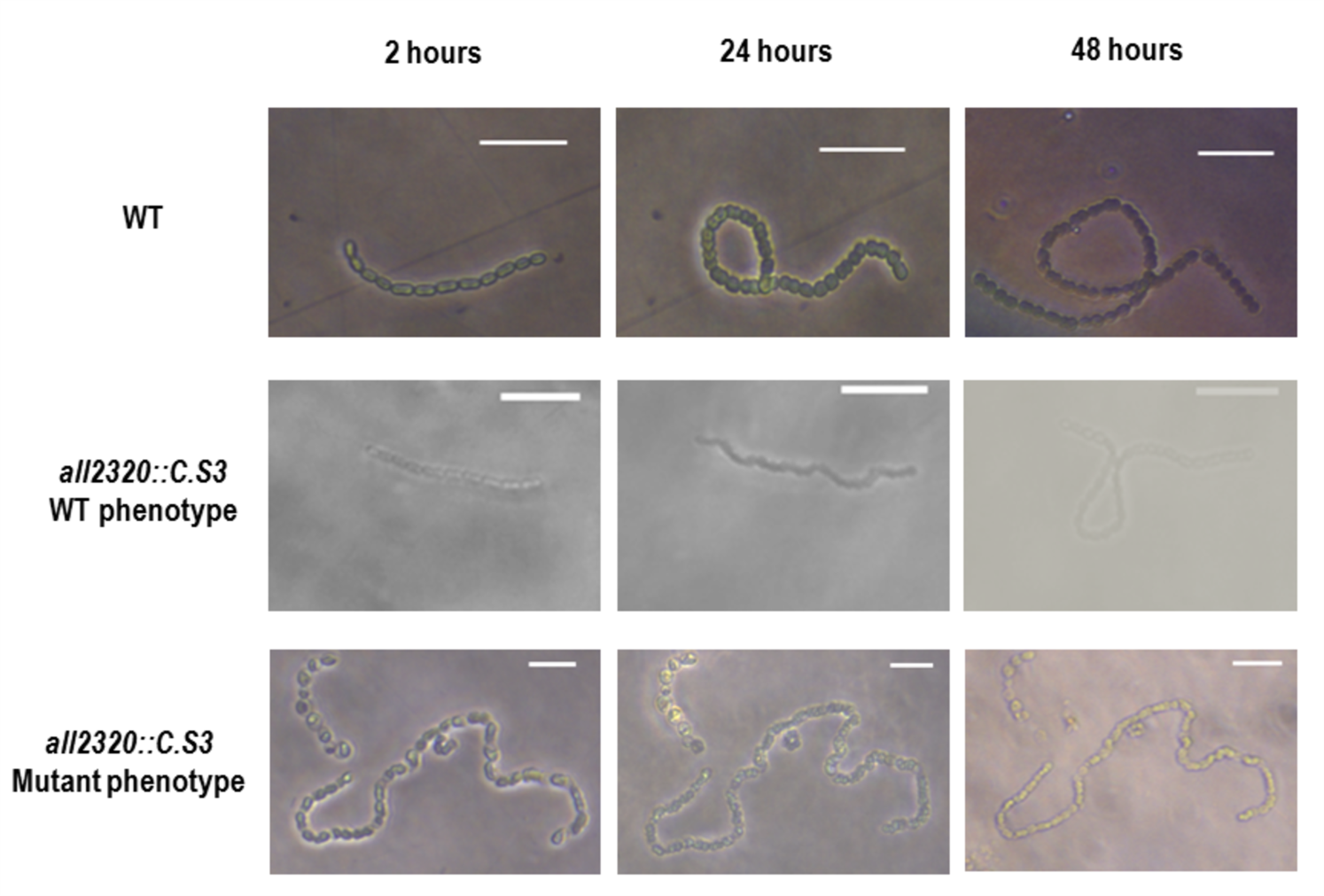
**

**Supplementary Figure 4. Light Microscopy of *Anabaena* PCC7120 and *all2320::C.S3* strains.** *Anabaena* PCC7120 and *all2320::C.S3* strains were grown in solid agar BG11 medium (supplemented with Sm and Sp for *all2320::C.S3*). Some of the filaments were visualized at 2, 24 and 48 hours of growth. The two types of filament phenotypes (wild type (WT) and mutant) were observed for *all2320::C.S3.* Bar scale 20 μm.

## Supplementary Tables

**Supplementary Table 1.** Oligodeoxynucleotide primers used in this work. The restriction endonuclease sites incorporated are shown in bold.

Primer Sequence (5’ – 3’)

________________________________________________________________________________________

all2320-1 CTCATCGCCGTAGCCAAT

all2320-2 **GCTAGC**ACCTCCACCGCCACGACTTTGGTGTTCAAATAAAG

all2320-3 CATCTAACTTGTC**ATCGAT**TTGTGTTGCT

all2320-4 AGCAACACAA**ATCGAT**GACAAGTTAGATG

qrnpB F TCTTGGTAAGGGTGCAAAGG

qrnpB R GCGGAACTGGTAAAAGACCA

qall2320 F GTCTGCCTCAAAAGCAAAGG

qall2320 R GTTCAAATAAAGGGCGGACA

qhglD F TCCACCCACGATTAATGTCA

qhglD R CGCGTGTTTAGTTCCGGTAT

qftsZ F TGTATCTGCTCCCCCAAAAC

qftsZ R CGCCGTCTCTGAAGAAAATC

all2320extCS3F CCTCCTCCGTACCCCTGTA

CS3rev CGGCCTTGCTGTTCTTCTAC

_________________________________________________________________________________________
