# Supplementary figures and images for: CyDiv, a Conserved and Novel Filamentous Cyanobacterial Cell Division Protein Involved in Septum Localization
Source: Front Microbiol. 2016 Feb 10;7:94. doi: 10.3389/fmicb.2016.00094 (PMC4748335; doi:10.3389/fmicb.2016.00094)

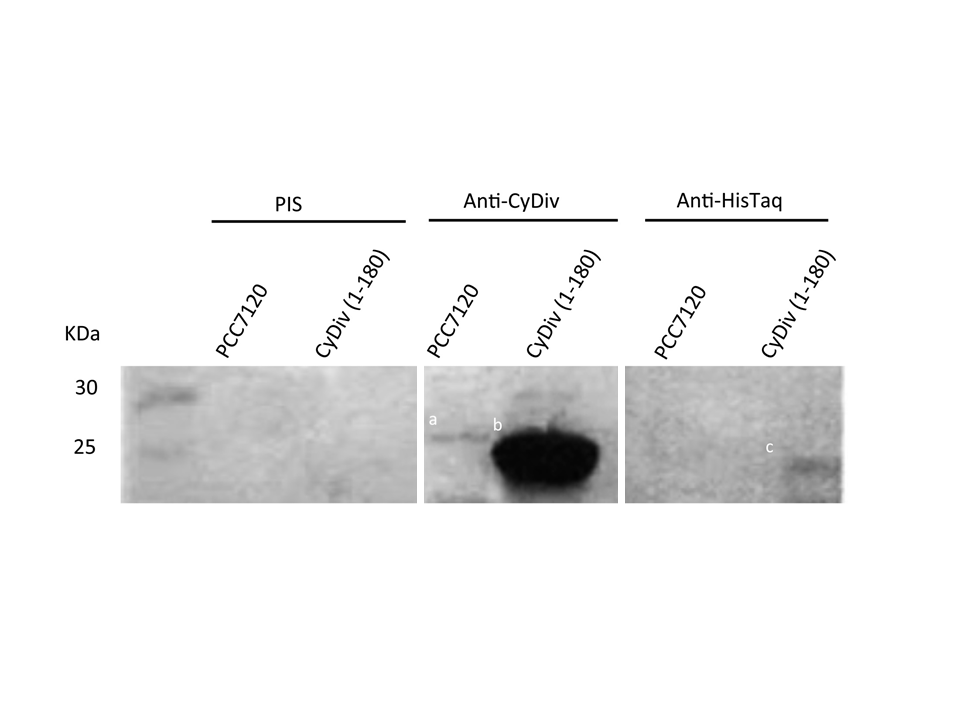

Supplement: Supplementary file 2 [file Image_1.TIF]

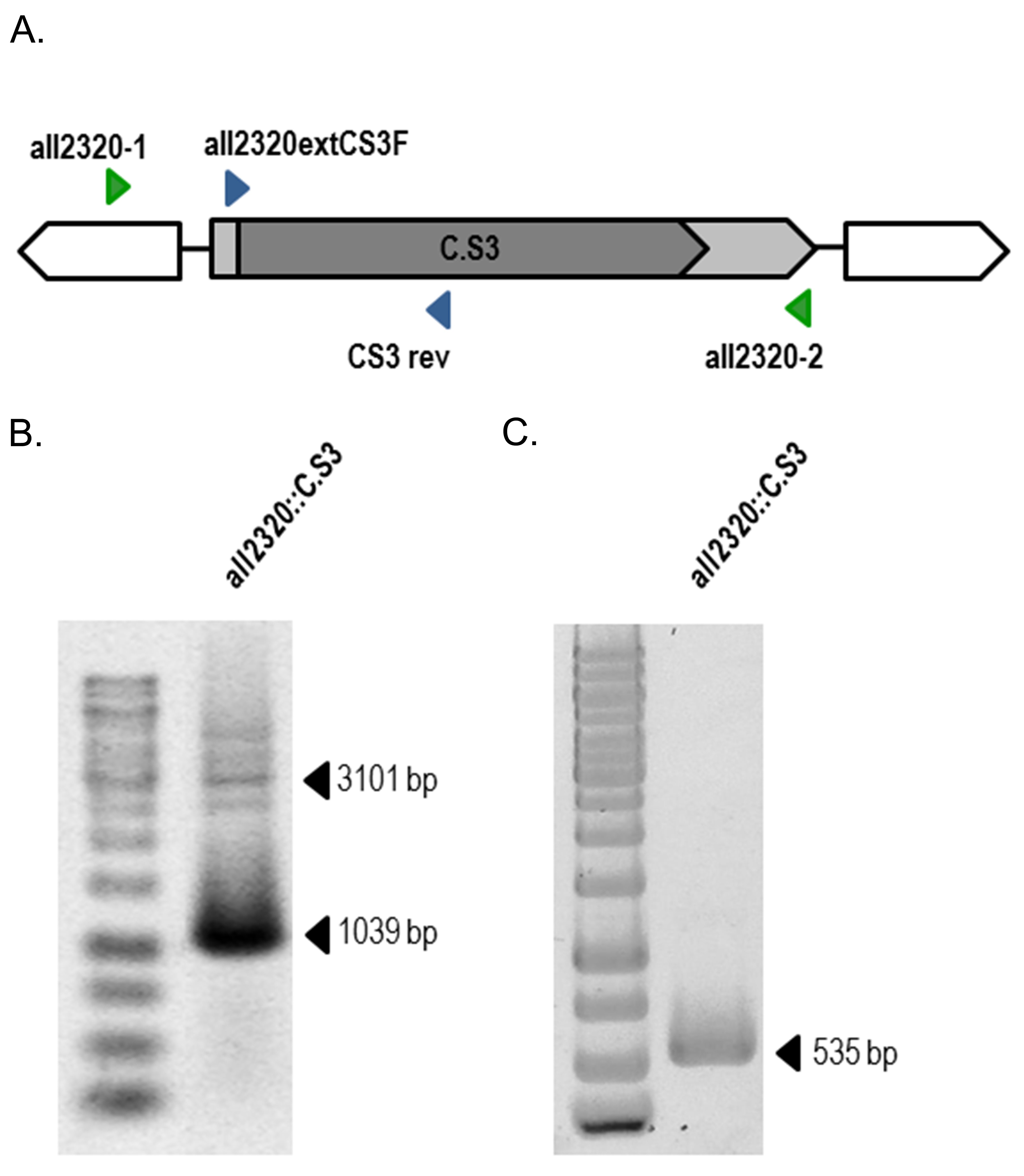

Supplement: Supplementary file 3 [file Image_2.JPEG]

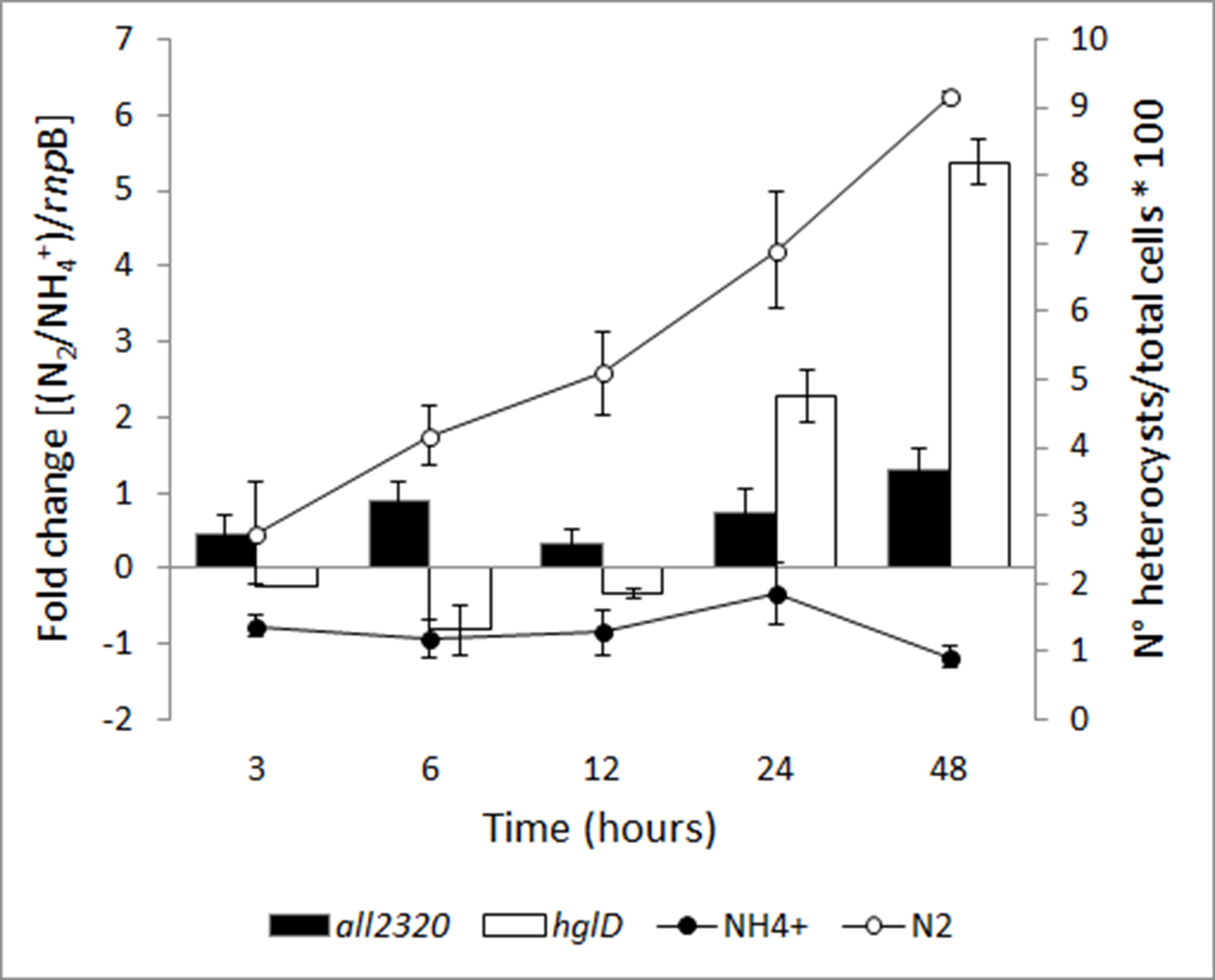

Supplement: Supplementary file 4 [file Image_3.JPEG]

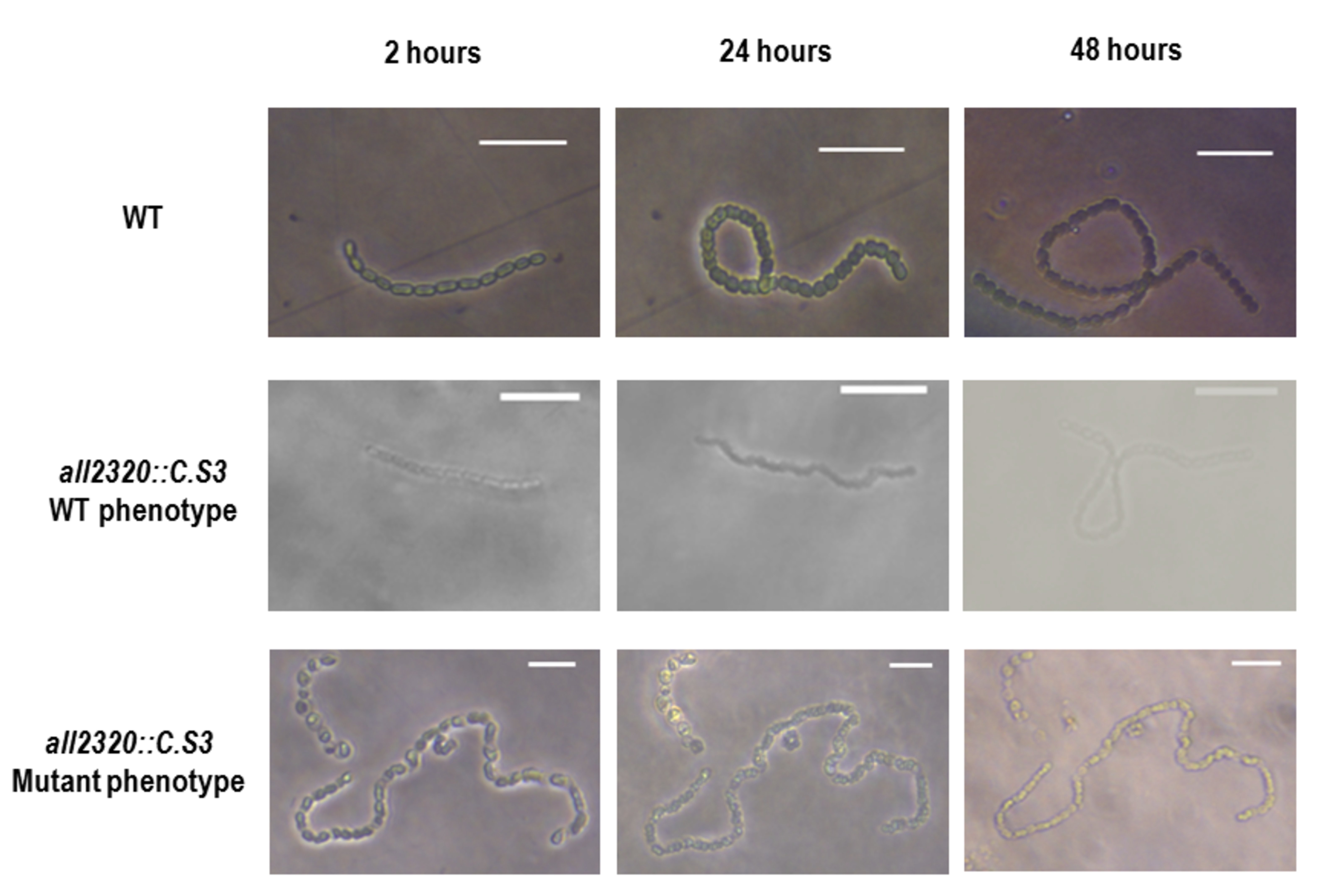

Supplement: Supplementary file 5 [file Image_4.JPEG]
